# The Rosewell Park Medical Club

**Published:** 1908-04

**Authors:** George F Cott


### The Rosewell Park Medical Club

Reported by GEORGE F COTT, M. D., Secretary.

THE fifth meeting of the Roswell Park Medical Club was held March 4, 1908, at the residence of Dr. James E. King. After dinner, reading of minutes having been suspended, Dr. William C. Krauss was presented and read a paper, by invitation, on Spinal Cord Tumors. The paper was illustrated, showing how nerves, motor and sensory, were affected by spinal cord tumors. He stated that during a period of four years he collected eighty-six cases and later added thirty-five more with operation, all the cases reported up to 1903. We knew about spinal cord tumors clinically for Leyden of Berlin and Erb of

Heidelberg, foresaw surgical procedures for their removal. Nobody ventured to operate on these cases, however, until Gowers induced Horsely to operate on his case in London in 1888, which was really the first operation. On June 9, 1887, a fibromyxoma, intradural, was removed, and the patient recovered. Bazy had one case November 9, 1886 a Frenchman, age 45, who died three days later of nephritis, but was not reported until 1889.

In 1885, McEwen and 1887, Braus of Germany, also operated for paraplegia due to Potts disease, but not for tumor. Oppenheim reported nine out of fifteen cases operated upon as having been correct in diagnosis, thus showing the difficulty of diagnosis.

May, 1888, Abbe of New York, was the first American to operate for cord tumors. In 1904, Dr. Krauss reported three cases of psuedo tumors which presented all the symptoms of spinal cord tumors, but which finally all vanished suddenly. Hysteria, or a localised serous meningitis was the cause. He also reported a case of myeloma, of which only eight cases are on record; operation and recovery.

In 1892, Dr. Park operated upon a case for Dr. Krauss at the General Hospital. The man lived four months and twenty days. Tumor located at the third cervical segment, the highest point of any case at that time yet found to be operated and live. Died of recurrence. This was an intradural round cell sarcoma, the size of the tip of the little finger. In 1903, Dr. Park operated upon another patient for Dr. Krauss for an intraspinal cyst; removed the cyst and the patient made a very good recovery. Seventeen cases of cysts are on record[1].

In America up to 1903, thirty-three cases of tumors were reported out of a total of eighty-six, twenty extradural, thirteen intradural and three intramedullary.

Forty per cent. recovered or were relieved of cord symptoms, nine improved, ten died at once or within a day or two after operation. These tumors are nowhere so benign as in the cord; not so in the brain, probably due to limited blood and lymph supply. ("Sarcoma of the eye, if early operation, always recovery;" Dr. Bennett).

In only three cases was a second operation necessary to find the tumor. These tumors occur twice as often in males as in females and most often between thirty and forty years. In seven cases accident was the cause of growth.

Important symptoms of cord tumors are: pain, slowly progressive motor paralysis, accompanied by anesthesia, exaggerated

1. A third case was diagnosed by Dr. Krauss and operated by Dr. McGuire, at Buffalo General Hospital, early in March, 1908, and found to have been an intramedullary tumor. Patient died shortly after.

tendon reflexes, local tenderness over the spine. Sherrington's multiple distribution of nerve supply from the posterior spinal nerves, bladder and rectal paralysis. The symptoms, of course, depend upon the segment of the cord affected.

After a short discussion Dr. Bentz followed with a paper on, Ophthalmic Tuberculin Test. This was first used by him January 19, 1807, using a ½ to 1 per cent. solution, dropping it in the eye, producing hyperemia in infected individuals. Irritation lasts three to four hours and six to ten hours, and clears up in two or three days.

Dr. Russell tried the ophthalmo-tuberculin fluid test in his own case using Parke, Davis & Company's fluid with positive reaction, and again using as much as one-fourth centigram with no reaction at all.

Dr. Potter said the case of cystitis, reported by Dr. Bentz, gave the positive reaction with the ophthalmo-tuberculin fluid. If we can diagnose typhoid fever in every early case we shall have gained a great step in advance. One case recently had sore throat, which improved suddenly, but temperature kept up. Typhoid was determined at the end of two weeks.

Dr. Bentz reported that Dr. Bolden got 93 per cent. positive results with blood culture, while the Widal test gave but 90 per cent. in the first weeks of the disease. The tuberculin reaction was not reliable in children under two years of age. Scarification and rubbing in tuberculin was practised in Germany and produced good results.

Dr. Bentz in closing said he was unable to explain the discrepancy between his results with the opthalmo-tuberculin test and those of Dr. Russell.

Dr. King reported for Dr. McKenney a case of narcolepsy.

Dr. King showed a specimen of enlarged and somewhat hardened thymus gland of a child seven months old. Child well until day before death, when it had very little temperature. At half-past four child seemed healthy, but half-past seven became blue with labored respiration and died shortly after. Post-mortem showed large thymus and very large lymph nodes throughout the intestines.

Dr. Russell stated that in case of status lymphaticus nodes are large throughout the body, this stimulates infection which kills quickly and can be introduced by injecting antitoxin which may cause rapid degeneration and in that way explain those occasional sudden deaths in diphtheria.

Dr. Gibson proposed a vote of thanks to Doctors Krauss, Bentz and McKenney for their valuable contributions, which was unanimously adopted.

